# Does Cataract Surgery Simulation Correlate with Real-life Experience?

**DOI:** 10.4274/tjo.10586

**Published:** 2018-06-29

**Authors:** Ayşe Bozkurt Oflaz, Bengü Ekinci Köktekir, Süleyman Okudan

**Affiliations:** 1 Selçuk University Faculty of Medicine, Department of Ophthalmology, Konya, Turkey

**Keywords:** Surgical training, phacoemulsification surgery, virtual reality simulation

## Abstract

**Objectives:**

To evaluate the correlation of cataract surgical simulator and real-life surgical experience and its contribution to surgical training.

**Materials and Methods:**

Sixteen doctors in our department were divided into three groups based on their surgical experience. After being familiarized with the device, the participants were evaluated while performing the navigation, forceps, bimanual practice, anti-tremor and capsulorhexis stages. The capsulorhexis stage was repeated five times. Participants were also assessed while performing capsulorhexis again with their non-dominant hand. The influence of repetition and surgical experience on the recorded points was evaluated. P values below 0.05 were considered statistically significant.

**Results:**

There was correlation between the participants’ surgical experience and their scores in the capsulorhexis module. Their dominant hand was more successful than the non-dominant hand in capsulorhexis (p=0.004). Capsulorhexis scores increased with repetition (p=0.001).

**Conclusion:**

Results achieved with the cataract surgery simulation device correlate with surgical experience. The increase in performance upon repeated practice indicates that the simulator supports surgical training.

## Introduction

Surgical training with simulators is utilized in many branches because it allows training in a controlled environment with objective assessment of progress. Surgical simulation also has potential as an important part of the surgical training of ophthalmology residents. Although the number of surgical procedures performed on actual patients is important, it has been proposed that computer-based surgical simulation training will increase success and reduce complication rates in real surgeries.^1^

Cataract surgery is one of the most common surgical procedures in ophthalmology.^2^ The procedure requires good hand-eye coordination and has a long learning curve.^3^ Numerous studies indicate that simulator and wet-lab training increase surgical performance, shorten residents’ learning curve and reduce physician-related complications.^1^

Three simulation devices have been developed for use in cataract surgery: Eyesi^®^ (VRmagic, Mannheim, Germany), PhacoVision^®^ (Melerit Medical, Linkoping, Sweden) and MicrovisTouch^®^ (ImmersiveTouch, Chicago, USA). Most of the studies published in the literature utilized the Eyesi^®^ simulator.^1^ This device has been reported to provide systematic, effective and reliable surgical training at a lower cost.^4^ There are few studies on the MicrovisTouch^®^ and PhacoVision^®^ simulators.^1^ Distinguishing features of the MicrovisTouch^®^ are the advantages of receiving tactile feedback and having an adjustable virtual head. However, this device only has a capsulorhexis stage and not the other modules available in the Eyesi^®^ simulator.^1^

The cataract surgery simulator in our clinic (Eyesi^®^) is used regularly in surgical training to facilitate the transition to practical application. 

The present study was designed to determine the extent to which simulated procedures contribute to cataract surgery training and correlate with real-life experience.

## Materials and Methods

After obtaining approval from the institutional ethics committee, the physicians working as residents in our clinic were informed about the nature of the study and they provided informed consent to use their scores in the study. Sixteen physicians were separated into three groups according to their surgical experience. Group 1 included 7 residents with no experience in cataract surgery who had been working for 2-10 months. Group 2 comprised 6 residents who had performed 20-80 cataract surgeries and been working for 12-24 months. Group 3 included 3 faculty members with experience of 1000-1500 cases. Each physician underwent ophthalmologic examination and those with best corrected visual acuity of 20/20 in both eyes, sufficient stereopsis and normal findings on slit-lamp examination were included in the study.

The study was conducted using the Eyesi^®^ surgical simulation device in our clinic. Only cataract surgery simulation software was installed on our simulator. 

All of the simulator sessions in the study were supervised by the same researcher (A.B.O.). The participants were first familiarized with the surgical simulator. They were then asked to perform the navigation application as the first stage, followed by the first steps of the forceps, bimanual application, anti-tremor module and capsulorhexis stages.

### Statistical Analysis

In the capsulorhexis module, participants were asked to perform the same procedure twice, first with their dominant hand and then with their nondominant hand. The capsulorhexis procedure was repeated four more times using the dominant hand. Finally, the third stage of the capsulorhexis module, “capsulorhexis in mature cataract”, was performed and the participants’ scores were noted. 

SPSS 15.0 software was used for statistical analysis of the study data. A nonparametric correlation value between surgical experience and the simulator scores was determined (Spearman correlation coefficient). Other data were analysed nonparametrically using Kruskal-Wallis test and p values below 0.05 were considered significant.

## Results

**Figure 1:**
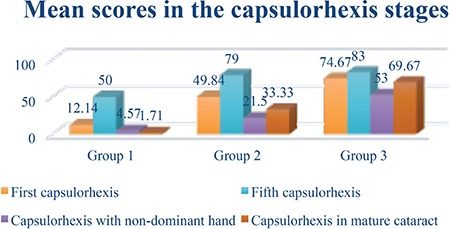
Capsulorhexis stage scores of the groups

Seven of the 16 physicians in the study were female, 9 were male; the mean age was 30.18 years. Simulator scores for the capsulorhexis stage in both dominant and nondominant hands were positively associated with the number of real procedures performed (Figure 1). 

Capsulorhexis performed with the dominant hand was more successful than capsulorhexis by the nondominant hand (p=0.004). The success of capsulorhexis increased with repeated attempts (p=0.001) (Figure 1). 

The groups of physicians with less experience exhibited sharper increase in success with practice. The “capsulorhexis in mature cataract” stage was completed more successfully by group 3, who had the most practical experience (Figure 1).

When the groups’ scores were analyzed in comparison with their experience using the Kruskal-Wallis test, the more experienced group was found to have significantly different scores than the less experienced groups (p=0.009).

According to Spearman correlation analysis, capsulorhexis scores correlated with surgical experience at all stages (Table 1).

## Discussion

Increasing interest in surgical simulators in recent years has inspired many studies investigating the contribution of these devices to surgical practice and their consistency with real life. In ophthalmology practice, training courses are conducted using these devices. This gives physicians the opportunity to receive theoretical and practical training in cataract surgery or vitreoretinal surgery.

**Figure 2:**
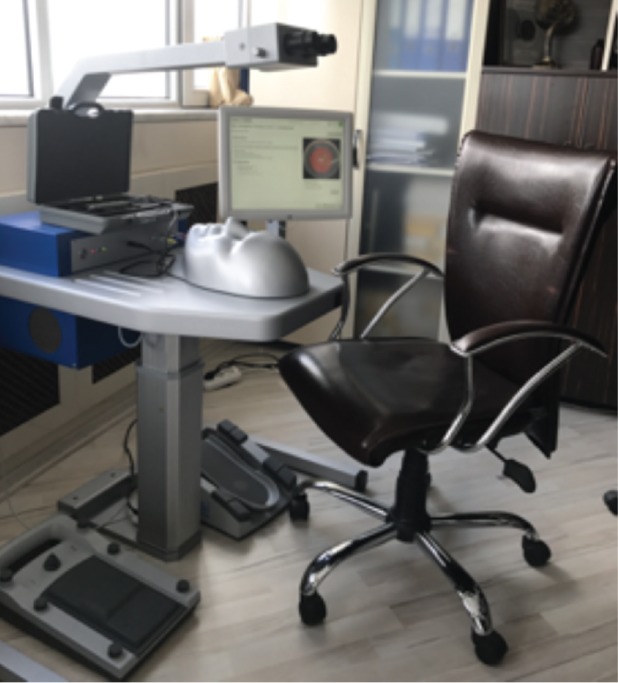
The cataract surgery simulator device used in our clinic

The Eyesi^® ^simulator has been designed with a binocular vision system that enables adjustable depth and magnification with a pedal-controlled imaging. In the model head, the right eye has ports in several axes (at 8, 6, 5 and 3 o’clock positions) to allow the users to handle the probes that simulate surgical instruments (Figure 2). 

In the various modules, while performing steps of varying difficulty, the users are scored by the system from 0 to 100 according to the time elapsed, eye deviation, trauma to tissues such as the cornea, lens and iris and whether the stage was completed successfully.

**Figure 3:**
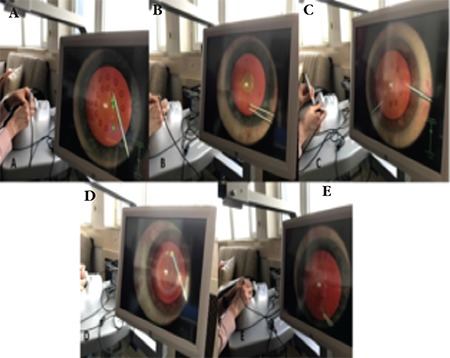
Screen view during the navigation (A), forceps (B), bimanual application (C), anti-tremor module (D) and capsulorhexis (E) stages of the simulator

In the navigation stage, the user must use the probe to touch spheres in the anterior chamber and turn them green. In the forceps module, the user is asked to bring triangular targets located at the edges into an area in the anterior chamber. In the bimanual application, the user must touch the spheres with the probes using both hands simultaneously. The anti-tremor module involves using the probe to push the sphere in a certain direction. In the capsulorhexis stage, the user applies viscoelastic material to the anterior chamber, uses a cystotome to create a flap and makes a circular capsulorhexis using forceps. In the ‘capsulorhexis in mature cataract’ stage of this module, the users can also use tissue dye (Figure 3). The following steps include grasping the lens, cracking and chopping the lens, irrigation and aspiration and inserting the intraocular lens. 

A study by Mahr and Hodge^5^ demonstrated the validity of the anterior segment anti-tremor and forceps training with the Eyesi^®^ simulator. Fifteen participants were divided into a group of 12 inexperienced surgeons and a group of 3 experienced surgeons. Experienced surgeons scored higher and completed the stages in a shorter time.

Banerjee et al.^6^ used the MicrovisTouch^®^ simulator to investigate the concurrent validity of capsulorhexis performance metrics (duration, number of capsular grasps per completed capsulorhexis and roundness of capsulorhexis) and found that simulator results correlated with real-life performance.

Selvander and Asman^7^ assessed the validity of the capsulorhexis, hydrodissection, phacoemulsification, navigation and forceps training stages in the Eyesi^®^ simulator. There were 24 participants in two groups: 17 medical students and 7 experienced surgeons. The experienced surgeons had statistically better scores in simulated capsulorhexis, navigation and forceps modules, while the difference was less pronounced in the phacoemulsification and cracking and chopping stages. The same researchers asked 35 medical students to repeat the stages in order to determine whether repeated practice and the previous stages affected the learning curve and they reported a steep learning curve for the first 10 attempts, followed by a plateau. They also reported concurrent validity of the capsulorhexis stage in the latter study.^8^

Privett et al.^9^ evaluated the validity of the capsulorhexis stage with Eyesi^® ^in a study including 23 participants, a group of 16 medical students and a group of 7 experienced surgeons. The participants’ scores and completion times for capsulorhexis were found to be correlated with real life.

Thomsen et al.^10^ tested the Eyesi^®^ cataract surgery simulator in 26 physicians with no cataract surgery experience, 11 experienced cataract surgeons and 5 vitreoretinal surgeons. They determined in this reliability and validity study that experienced cataract surgeons and vitreoretinal surgeons received scores that were adequate or higher. Our data also suggested that the scores obtained in the modules increased with surgical experience.

In another study, 63 participants including 31 medical students and 32 ophthalmologists were randomly divided into 2 groups. All participants were asked to perform capsulorhexis on porcine eyes at two time points. In the interval, one of the groups was trained in the capsulorhexis stage of the Eyesi^®^ simulator. Videos of the procedures were reviewed by an independent team who scored the participants’ performance. The group that underwent simulator training showed significant improvement in scores at the second time point and significantly higher scores overall compared to the control group. These findings support the contribution of simulation to surgical training.^11^

Bergqvist et al.^12^ also demonstrated that simulator scores increased with repeated practice and emphasized the contribution of this practice to training. We also observed in our study that the participants exhibited better performance when performing capsulorhexis for the fifth time. This finding suggests that repetition may contribute to surgical practice.

In a subjective evaluation based on users’ feedback, Dooley and O’Brien^13^ reported that capsulorhexis was the most difficult stage in the simulator and stated that allocating more time to this stage during stimulator practice may be beneficial for training.

Belyea et al.^14^ investigated the role of simulators in resident training by retrospectively evaluating 592 surgeries by 42 physicians (17 simulator-trained and 25 untrained) with regard to total surgery time and complication rates and found that surgeons with simulator training had a shorter learning curve. Simulator training was associated with lower rate and severity of surgical complications and shorter procedure times.

Pokroy et al.^15^ also demonstrated that the simulator is beneficial in surgical training and that practice shortened surgery time. In a study investigating the efficiency of a training program established by the International Ophthalmic Simulation Forum using the Eyesi^®^ simulator, Saleh et al.^16^ compared the pre- and post-training simulator scores of 16 inexperienced surgeons. They showed that scores in all stages increased significantly and there was a particularly important impact on the learning curve in the first year of surgery. In our clinic, practicing with the Eyesi^®^ became routine when learning the stages of cataract surgery and preparing for initial real-life procedures and we found that this practice increased surgical safety.

Sachdeva and Traboulsi^17^ observed a significant difference in performance when they compared participants with insufficient stereopsis with a control group. This was not taken into consideration in our study because all of the ophthalmology residents had normal stereopsis. Still, the fact that insufficient stereopsis influences performance is evidence of the validity and reliability of simulation.

Besides their role in training, simulators are also ideal to evaluate the effect of surgical environment on surgeon performance. Most of these studies cannot be conducted during real procedures due to ethical concerns related to patient safety. Simulators have been used to evaluate how surgical performance is affected by tiredness, visual acuity, use of the nondominant hand, surgeon distraction and the use of beta-blockers.^18,19,21,22^

During initial surgical experiences, the patient may be an unforgiving teacher. It is predicted that simulators will become more common in daily practice to enhance the learning of residents early in their careers. Although there are foreign publications regarding the role of simulators in virtual reality studies and training, there are no published studies in this area in Turkey. Therefore, our aim was to raise awareness of this topic by sharing our clinical experience. 

## Conclusion

The scores obtained in the capsulorhexis stage show that the cataract surgery simulator is correlated with real life. The association between repeated practice and improved performance indicates that the device facilitates training.

Simulators may find a place in practice because they allow trainers to explain aspects of the surgical technique to inexperienced trainees without time constraints and the trainee can freely observe the technique in question. Because real patients are not involved in the procedure, simulators provide a less stressful and more convenient environment both for trainees and trainers. 

Performing the procedure first in the simulator and then on real patients may be more ethically appropriate. It instills self-confidence in the trainee before operating on actual patients and helps prevent some of the potential medicolegal problems. In short, simulator training is ideal for physicians to foster surgeon confidence prior to real surgical procedures and prevent possible complications.

## Figures and Tables

**Table 1:**
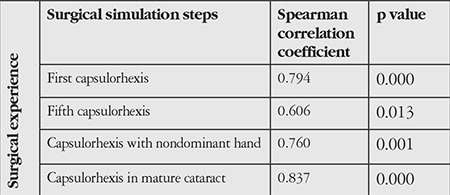
Comparison of simulator scores according to surgical experience
